# ChIP and Chips: Introducing the WormPharm for correlative studies employing pharmacology and genome-wide analyses in *C. elegans*

**DOI:** 10.14440/jbm.2016.109

**Published:** 2016-06-10

**Authors:** Jung H. Doh, Andrew B. Moore, İrem Çelen, Michael T. Moore, Chandram R. Sabanayagam

**Affiliations:** ^1^University of Delaware, Delaware Biotechnology Institute, Newark, DE, USA; ^2^University of Delaware, Department of Biological Sciences, Newark, DE, USA; ^3^University of Delaware, Center for Bioinformatics and Computational Biology, Newark, DE, USA

**Keywords:** *C. elegans*, WormPharm, Wormchip, CeHR, axenic, growth kinetics, ChIP-seq

## Abstract

We present the WormPharm, an automated microfluidic platform that utilizes an axenic medium to culture *C. elegans*. The WormPharm is capable of sustaining *C. elegans* for extended periods, while recording worm development and growth with high temporal resolution ranging from seconds to minutes over several days to months. We demonstrate the utility of the device to monitor *C. elegans* growth in the presence of varying doses of nicotine and alcohol. Furthermore, we show that *C. elegans* cultured in the WormPharm are amendable for high-throughput genomic assays, *i.e.* chromatin-immunoprecipitation followed by next generation sequencing, and confirm that nematodes grown in monoxenic and axenic cultures exhibit genetic modifications that correlate with observed phenotypes. The WormPharm is a powerful tool for analyzing the effects of chemical, nutritional and environmental variations on organism level responses in conjunction with genome-wide changes in *C. elegans*.

## INTRODUCTION

In the laboratory, *C. elegans* are maintained on an Escherichia coli (*E. coli*) food source, either on nematode growth media (NGM) agar plates or in S-media liquid cultures [1, 2]. Other methods of cultivation employ axenic media, which were not often used for maintaining *C. elegans* with the exception of transport into space [3]. A number of axenic liquid media have been developed for culturing of *C. elegans* and other nematodes [4-9] since the 1950’s and most of these media were chemically defined in which all of the chemical components are known. The development of a defined medium, the *C. elegans* Maintenance Medium (CeMM) was introduced by Lu and Goetsch (1993) [8]. However, animals cultured in CeMM took considerably longer (2-3 times) to develop compared to worms grown on *E. coli* seeded NGM plates. In order to enhance development and reproduction in chemically defined media, Clegg *et al*. (Clegg *et al*. 2004, unpublished data) formulated the *C. elegans* Habitation and Reproduction medium (CeHR), which was used by the U.S. Army to assess the direct effects of environmental, nutritional and chemical variations on *C. elegans*. CeHR uses a base medium similar to CeMM but includes 20% ultrahigh-temperature pasteurized skim milk for additional nutrients in order to improve development and fecundity; however, the skim milk in CeHR no longer makes it chemically defined.

Using axenic media for worm cultivation has many advantages over the more widely used *E. coli*-based media. Axenic media allows for clean, non-contaminated extract preparations for genetic assays and it is optically clear for microscopy observations. Axenic media can be stored for months and readily supplied to *C. elegans*, whereas *E. coli* cultures generally have to be manually replenished within several days to maintain a fresh food supply [10]. Axenic media can easily be supplemented with chemical compounds or drugs allowing for media versatility for pharmaceutical studies. Monoxenic media typically cause variability in pharmaceutical assays because the bacteria may metabolize the drugs or catabolize organic compounds. CeMM fed *C. elegans* have been shown to exhibit about a two-fold increase in lifespan and can be cultured for multiple generations [9, 11, 12], which enable long-term, multi-generational studies. Worms grown in bacteria supplied S-media generally develop faster but longevity and viability of subsequent generations are drastically compromised [2]. Studies have suggested that worms can die during development in *E. coli* cultures because the bacteria deplete the oxygen in the environment [13, 14]. *E. coli* also release deleterious toxins that are harmful to *C. elegans* [15-17]. Lastly, axenic media can be utilized in microfluidic systems without bacteria forming biofilms compromising the device.

In the past decade, scientists have developed a number of sophisticated microfluidic techniques to identify microscopic phenotypes associated with environmental perturbations [18-30]. Recently, Xian *et al*. (2013) reported the development of the WormFarm, an integrated microfluidic device consisting of eight separate chambers, each capable of sustaining up to 50 nematodes [10]. The group describes computational algorithms to quantitate survival and age-related phenotypes from images and videos, and by feeding the worms bacteria on a daily basis, they are able to culture and track the nematodes throughout their lifespans. However, the bacteria cultures were manually changed every day preventing automation of the WormFarms. Despite the variety of microfluidic devices reported to date, none of them provide a means to maintain *C. elegans* for multiple generations or cultivate a sufficient number of animals for downstream biochemical assays.

Genome-wide assays involving biochemistry followed by next generation sequencing offer tremendous opportunities to obtain a comprehensive understanding of the genetic effects under environmental changes. A popular assay used to study these effects is chromatin-immunoprecipitation followed by next generation sequencing (ChIP-seq) [31]. While the range of applications for ChIP-seq is broad, the more common applications include characterization of DNA-binding transcription factors, the distributions of chromatin-modifying machinery, histone variants, and histone modifications. Most of these processes are dynamic in that they can alter gene expression in response to stimuli within a single generation and be passed on to subsequent generations [32, 33]. ChIP-seq in this sense is ideal for analyzing how organisms adapt or respond genetically to environmental changes in a relatively short period of time. However, one of the biggest limitations of ChIP-seq is that a substantial number of cells are needed to perform the experiments. Therefore, treating, culturing and harvesting cells in large enough quantities can be quite arduous and sometimes impractical.

We present the WormPharm, a computerized *C. elegans* micro-cultivator designed for ChIP-seq experiments, one of the most material demanding high-throughput genetic assays. We show that the sequence data from the ChIP-seq assays are of high quality and confirm that genetic variations arising from different growth environments correlate with observed phenotypes. Furthermore, the utilization of CeHR in the WormPharm facilitates long-term and multigenerational studies, and we demonstrate that the WormPharm can monitor organism level responses to chemical or environmental perturbations. We propose that the WormPharm will be a valuable tool in analyzing the effects of chemical compounds and environmental changes on behavior, development and genome-wide fluctuations on *C. elegans* biology.

## MATERIALS AND METHODS

### Nematode culture and strains

Standard procedures for nematode culture were followed with growth at room temperature unless otherwise stated. The strains used in this study were wild-type N2 and AB1 obtained from Caenorhabditis Genetics Center (CGC).

### Wormchip fabrication

The Wormchips were fabricated with polydimethylsiloxane (PDMS) (Dow Corning Corp, Sylgard® 184 Silicone Elastomer Kit). Acrylic molds were designed using the CorelDraw X6 software, and cut using a hobby laser (Full Spectrum Laser HL-40). The PDMS molds were heated and cured at 90°C for at least 4 h. Two holes at 90° angles from each other were made with a 2.0 mm biopsy punch (Robbins), and silicon tubes (Scientific Commodities Inc., Cat# BB518-40) were inserted into the holes to function as inlet and outlet points. The assembly was incubated in 100% ethanol overnight and the silicon tubes were sealed to the Wormchips with marine epoxy. Microscope coverslips (Warner Instruments, Cat# 64-0715) were then covalently bonded to the open face of the Wormchips via a plasma cleaner (Harrick Plasma PDC-32G). The Wormchips are exposed to an ultraviolet-ozone system (Novascan PDS Pro) at 50°C for 40 min and blocked overnight with a solution of 3% bovine serum albumin (Fisher Sci., BP1600) and 3% polyethylene glycol (Polysciences, Inc., Cat#16861). To construct agar OP50 NGM Wormchips, 1 ml of heated (55°C) NGM agar was poured to an acrylic mold with the exact dimensions as the Wormchips designed for CeHR and allowed to solidify. *E. coli* OP50 were seeded onto the agar and grown overnight in 37°C. Embryos were introduced to the OP50 NGM Wormchips and covered with a glass coverslip to prevent the media from drying out.

### The assembly of the WormPharm

The CeHR medium was adopted from an original recipe formulated by the US Army Center for Environmental Health research (Clegg *et al*. 2004, unpublished data). All worm cultures in the CeHR were started with embryos isolated from gravid adults grown on OP50 NGM plates via the bleaching protocol [1]. Approximately 1000 embryos were suspended in CeHR and pipetted into the Wormchips. The Wormchips were then assembled onto the WormPharm platform. The platform consists of a fluid pump system and a lab built microscope. A peristaltic pump (SimplyPumps PM100N) was used to pump CeHR from a cooled reservoir toward a six channel manifold (Western Analytical, P-152) and into six Wormchips. The outlet tubes of the Wormchips were connected to a second six channel manifold that was connected to a waste reservoir. Silicon tubes (Scientific Commodities Inc., Cat# BB518-40) and straight through tubing fittings (Value Plastics, AA-2) were used to connect the Wormchips to both reservoirs. The pumping rate is controlled via an Arduino Nano with ATmega328 microcontroller. A stepper motor was programmed to rotate six Wormchips in the visible frame of a microscope. The structural components of the pump system were constructed out of acrylic. The microscope unit is comprised of a transmitted LED light source, a diffuser, an optical lens (Nikon AF-micro Nikkor 60 mm macro lens), and a CMOS camera (Point Grey, FL3-U3-88S2C-C0).

### Growth kinetics assays

Equivalent amounts of embryos were introduced onto OP50 NGM or CeHR filled Wormchips. Images were captured using the WormPharm every 30 min over the course of four or six days for OP50 NGM or CeHR, respectively. All images and movies were taken with the FlyCapture software (Point Grey). Worms appear as dark objects with our transmitted illumination scheme. For the nicotine and alcohol assays, images were captured every 24 h for six days. Total dark pixels were measured from frames captured as 16-bit images. Background subtraction and thresholding were processed with the ImageJ software [34]. The upper thresholds levels were set at 20,000 for images of OP50 NGM plates and 60,000 for images of CeHR supplied Wormchips. Total dark pixels are proportional to the total worm mass.

### Nicotine and alcohol assays

Embryos were resuspended in CeHR (control) and CeHR with final concentrations of 1 mM, 10 mM and 20 mM nicotine (Sigma, N3876) and 200 mM and 400 mM alcohol (200 proof ethanol). 1 mM nicotine and 200 mM ethanol concentrations were administered for synergy experiments. Nematodes were observed for six days and movies and images were taken at 24 h increments.

### Nuclear extract preparation

Approximately 100,000 synchronized late-L4/early adult staged *C. elegans* were harvested from the CeHR supplied Wormchips, OP50 S-media supplied Wormchips and OP50 NGM plates. Worms were cleaned by sucrose floatation and homogenized with a Dounce homogenizer. Worms were immediately fixed and pulverized, and nuclear extracts were prepared and sonicated using the truChIP™ Tissue Chromatin Shearing Kit with SDS Shearing Buffer kit (Covaris®) with the following modifications. Animals were fixed with 100 μl of 1% methanol-free formaldehyde solution (Thermo Scientific, 28906) for 6 min and quenched using 58 μl of Covaris Quenching Buffer for 5 min. Samples were washed using the Covaris protocol and pulverized using the Covaris CyroPrep instrument twice at setting 5. Nuclear extracts were prepared according to the manufacturer’s protocol and sonicated using the Covaris S2 Adaptive Focused Acoustic Disruptor (Covaris®) at the following settings: Duty Cycle at 20%, Intensity at 5, Cycles per Burst at 200, and processing time of 13 min. All other settings were set as default.

### Chromatin immunoprecipitation and Illumina sequencing

Each ChIP was prepared in 500 μl of FA buffer (50 mM HEPES/KOEach ChIP was prepared in 500 μl of FA buffer (50 mM HEPES/KOH pH 7.5, 1 mM EDTA, 1% Triton X-100, 0.1% sodium deoxycholate, 150 mM NaCl with protease inhibitors). Five micrograms of anti-H3K27me3 antibody (MBL MABI0323) and 300 µg of extracts were rotated overnight at 4ºC. Ten percent of the extract was saved as a reference. 40 μl of blocked and washed magnetic beads (Life Technologies: DynaBeads® M-280 Sheep anti-Mouse IgG) were added and the incubation continued for an additional 4 h. Beads were washed and eluted as previously described [35]. Crosslinks were reversed by incubating with 20 μg of Proteinase K overnight at 55°C. Input DNA was also diluted in 114 μl elution buffer and treated with ChIP samples. DNA was purified on Qiagen PCR purification columns. Sequencing libraries were prepared using 5 ng of DNA with the TruSeq® ChIP Sample Preparation Kit A (Illumina®) according to the manufacturer’s protocol. ChIP libraries were sequenced on an Illumina HiSeq 2500 instrument set to rapid run mode using single-end, 1 × 51 cycle sequencing reads as per manufacturer’s instructions.

### Quality control and analysis of sequenced data

Raw sequence data from three libraries were examined for quality control with the FastQC software (version 0.11.2) [36]. The reads were mapped to the reference genome (WBCel235), obtained from Ensembl along with the corresponding annotation files, by using Burrows-Wheel Aligner (BWA), version 0.7.4 [37, 38]. Model-based Analysis for ChIP-Seq (MACS), version 2.0, software [39] was employed for calling the peaks from the mapped reads. The peaks were visualized with the Integrative Genomics Viewer (IGV), version 2.1.9 [40]. The annotation of peaks were retrieved from Hypergeometric Optimization of Motif EnRichment (HOMER), version 4.2 [41]. The heatmap was created by using the DiffBind package in the R software [42]. The functions of the genes were acquired from Wormbase, version WS246 [43].

## RESULTS

### The Wormchips

**Figure 1:**
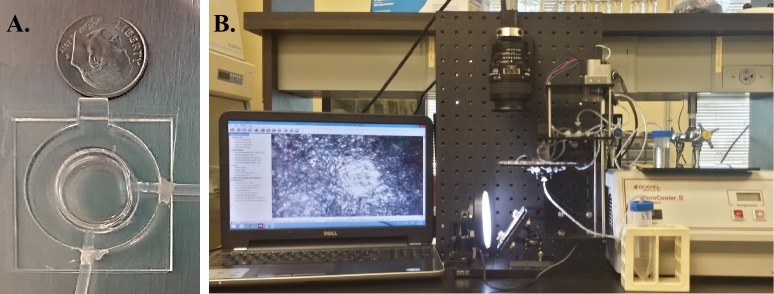
**The WormPharm**. **A**. The Wormchip. **B**. The WormPharm assembly consists of a fluid pump system supplying CeHR to six Wormchips rotating perpendicular to the field of view of the microscope. The microscope is connected to a camera for autonomous monitoring and recording worms on a computer.

Wormchips were developed for micro-cultivation of *C. elegans* in liquid media. They are constructed out of PDMS covalently bonded to microscope coverslips (**Fig. 1A**). The Wormchips are inert, non-toxic, non-flammable, optically clear, and designed to accommodate 1 ml of culturing media inside a 16 mm diameter by 5 mm thick cylindrical chamber. Oxygen exchange is permitted through a 200 µM layer of PDMS on the opposite side of the coverslip, and silicon tubes are connected at 90 degrees to the PDMS-coverslip assembly as inlets and outlets for the worms and liquid media. Wormchips were sterilized by ethanol and UV treatment and then blocked with a solution of polyethylene glycol and bovine serum albumin to prevent worms from sticking to the device.

### CeHR grown nematodes exhibit organism level phenotypes

**Figure 2:**
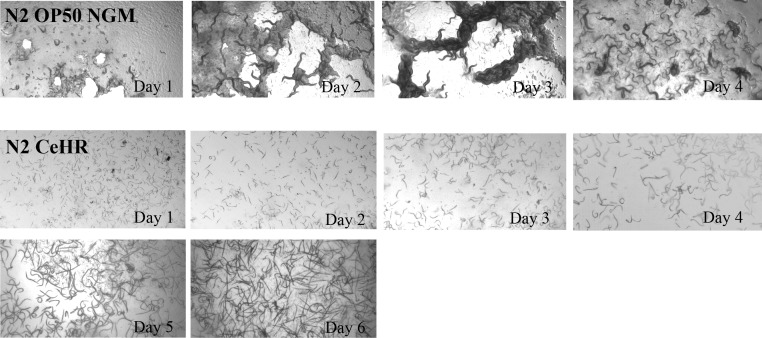
***C. elegans* development in the WormPharm**. *C. elegans* were grown on OP50 NGM agar (top) and liquid CeHR (bottom) Wormchips. Animals exhibit slower development and decreased fecundity in CeHR compared to OP50 NGM. *C. elegans* reach adulthood and produce offspring in approximately two days on OP50 NGM and four days in CeHR.

**Figure 3:**
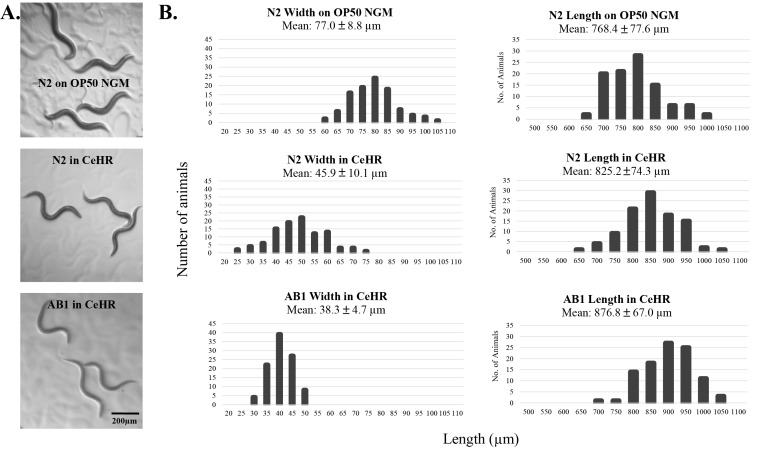
**C. elegans grown in CeHR display physical and crawling differences**. **A**. CeHR grown animals were placed onto OP50 NGM plates for comparison. N2 and AB1 adult worms grown in CeHR are longer, thinner and lighter in complexion compared to *C. elegans* grown on OP50 NGM. Crawling motion of CeHR grown N2 and AB1 adults is depicted by a 1.25 sigmoidal pattern instead of a 1.0 sigmoidal pattern observed in OP50 NGM grown adults. **B**. Histograms of the length and width measurements of 100 randomly-picked adult *C. elegans* cultured on OP50 NGM and in CeHR supplied Wormchips. On average, N2 adults on OP50 NGM are 768.4 ± 77.6 µm long and 77.0 ± 8.8 µm wide, N2 adults in CeHR are 825.2 ± 74.3 µm long and 45.9 ± 10.1 µm wide, and AB1 adults in CeHR are 876.8 ± 67.0 µm long and 38.3 ± 4.7 µm wide.

Previous studies have utilized CeHR to examine toxicology and nutritional requirements in *C. elegans* [44, 45]. Hunt *et al*. (2012) examined the effects of several heavy metals on morbidity/mortality and body morphologies (*i.e.,* vulva, intestine, and germ line) in *C. elegans* [45]. Hamza and colleagues utilized CeHR to report the requirement of heme for worm growth and development [44]. Others have established baseline phenotypes, *i.e.,* growth, development, fecundity and longevity, in nematodes grown in different chemically defined media [9, 11, 13, 14, 46]; however, studies establishing baseline data for *C. elegans* grown in CeHR is lacking. We assessed body morphology, rate of development, reproductive capability, and crawling behavior (once the worms are placed back onto agar plates) in CeHR grown animals (**Fig. 2** and **3**). CeHR cultured adult worms were 7.4% longer, 40.4% thinner, slightly lighter in complexion, and developmentally delayed compared to OP50 NGM grown animals. Furthermore, *C. elegans* in CeHR swims via a rapid C-shape thrashing motion instead of crawling via a sinusoidal pattern on bacteria seeded agar plates (OP50 NGM). When CeHR grown animals were placed back on to agar, the animals conformed a 1.25 sigmoidal crawling pattern instead of the 1.0 sigmoidal pattern typically depicted for animals grown on OP50 NGM (**Fig. 3A**). Lastly, a significant reduction in brood size was observed in CeHR fed nematodes. Growth of *C. elegans* in other chemically defined axenic media has previously been shown to result in decreased fecundity [9, 11]. Fecundity in CeHR was measured by qualitatively observing the offspring via the WormPharm and thus was not a quantitative assessment.

**Figure 4:**
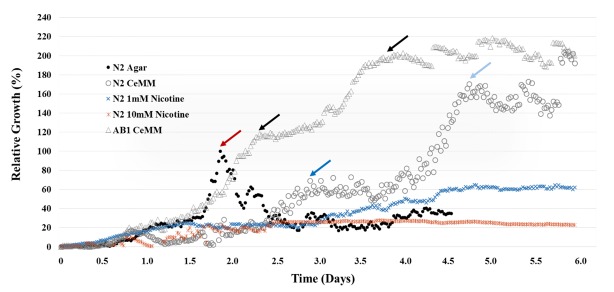
***C. elegans* growth kinetics in different growth conditions**. The WormPharm was used to measure the growth kinetics of *C. elegans* grown on OP50 NGM agar, in CeHR and in CeHR treated with 1 mM and 10 mM nicotine. On OP50 NGM agar, the N2 growth peaks at ~two days (red arrow). Afterwards, the growth decreases rapidly due to the depletion of the OP50 food source. In CeHR, the N2 growth exhibits a slower increase, reaching the first plateau at ~2.75 days (blue arrow) and a second plateau at ~4.75 days (light blue arrow). The AB1 growth displays a quicker increase than the N2 population in CeHR and also undergoes two waves of population increases (black arrows). Fluctuations in the growth kinetics are observed after the second plateaus for N2 and AB1 in CeHR due to large migrations of the worms in and out of the field of view. In the presence of 1 mM or 10 mM nicotine, the growth increase is significantly delayed. The plots represent relative growths of *C. elegans* grown in different conditions compared to OP50 NGM agar grown N2 animals with the highest peak set at 100%.

Natural wild-type strains of *C. elegans* have been isolated from numerous sites around the world [47] and these strains have been shown to display different traits. For example, progeny number and generation time under standard laboratory conditions vary among wild isolates [48, 49]. We asked if there is a difference in worm development and growth kinetics between two strains of *C. elegans*, the N2 lab strain and the natural isolate AB1 strain from Australia. We observed no significant differences in swimming and crawling behaviors between CeHR grown AB1 and N2 animals. However, AB1 adults were even longer, thinner, developed faster and produced more offspring than N2 animals in CeHR (**Fig. 3** and **4**).

### Autonomous cultivation of C. elegans in the WormPharm

The feeding of the worms and exchange of fresh CeHR was automated so that the WormPharm can function without human interaction. A fluid pump system was built to introduce a flow of 400 μl of CeHR per day from a cooled CeHR reservoir into six Wormchips (**Fig. 1B**). The Wormchips and the fluid pump system are assembled in conjunction with a lab built microscope. A camera connected to the microscope allows for autonomous monitoring of nematode populations via images or videos. To date, the longest period of time that the animals were autonomously maintained in the WormPharm was for four months. However, the length of time a WormPharm can maintain *C. elegans* can be controlled by varying the initial number of animals introduced into the Wormchips.

We measured the growth dynamics of *C. elegans* grown on OP50 NGM and in CeHR using the WormPharm (**Fig. 4**). The WormPharm was set to take images every 30 min over the course of four and six days for OP50 NGM agar and liquid CeHR supplied Wormchips, respectively. All frames were processed using the ImageJ software [34] to quantify the growth kinetics (Materials and Methods). On OP50 NGM, the animals grew to adulthood in ~2 days. After the depletion of the food source, animals started to burrow into the agar, resulting in an apparent drop in the growth kinetics (**Fig. 2** and **4**; *Movie S1*). To note, the drop in growth kinetics does not indicate mortality of the starved animals, but rather resulted from the inability to visually capture animals that burrowed into the agar soon after depleting the *E. coli* OP50. In CeHR, clumps of eggs are visible at the beginning of the experiment. After the first day, the egg clumps dissipate and larvae are present until the fourth day when new offspring are detected (*Movie S2*). Overall, development and growth were delayed, where the nematodes reached adulthood and the first wave of growth increase began to plateau around the third day. Shortly after, the next generation of L1 larvae were detected, followed by a second wave of growth increase that persisted until the end of the experiment (**Fig. 2** and **4**). The CeHR supplied WormPharm was able to autonomously sustain the animals over the course of the experiment and for multiple generations, which was unachievable in OP50 NGM.

### Utilization of the WormPharm to assess phenotypes associated with nicotine and alcohol

**Figure 5:**
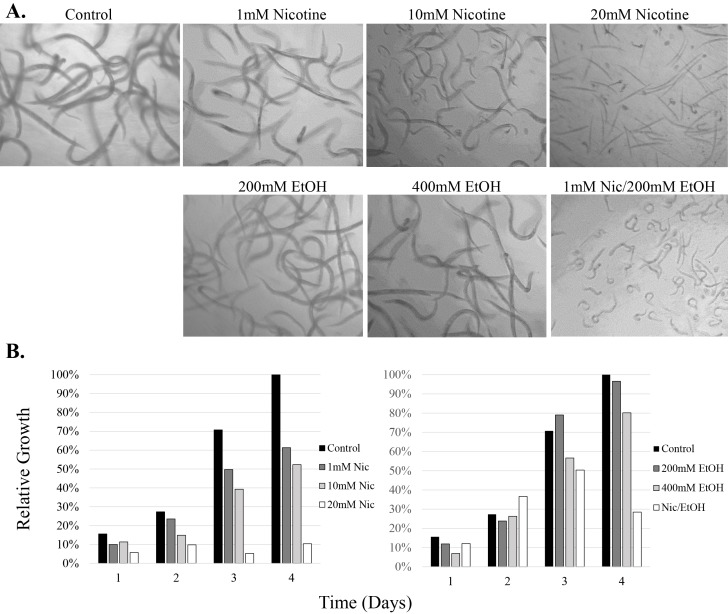
***C. elegans* display aberrant growth and development in response to nicotine and alcohol**. **A**. N2 animals were grown in CeHR (control) and CeHR treated with 1 mM, 10 mM or 20 mM nicotine (Nic), 200 mM or 400 mM ethanol (EtOH), and a combination of 1 mM nicotine and 200 mM ethanol. Images were taken after four days of treatment; refer to text and Supplemental **Figure 1** for more details. **B**. Quantitative growth comparisons of N2 animals grown in the presence of varying concentrations of nicotine and alcohol. The bars represent the relative growths of animals at one to four days of nicotine and ethanol treatments compared to the control (animals in untreated CeHR) on the fourth day set at 100%.

Assaying the effects of chronically exposing *C. elegans* to varying concentrations of ethanol and nicotine has been undertaken in previous studies utilizing NGM and a bacterial food source [50-55]. Ethanol studies regarding chronic exposure have shown that increasing concentrations of ethanol cause reductions in brood size and delay in development [50]. Nicotine has been observed to either stimulate or depress motor functions depending on concentration, and has effects on egg laying behavior [51]. We wanted to observe what effects certain concentrations of these drugs would induce in CeHR raised *C. elegans*, compared to prior studies utilizing NGM. The WormPharm was utilized to monitor *C. elegans* in the presence of 1 mM, 10 mM and 20 mM nicotine and 200 mM and 400 mM ethanol for over the course of four days (**Fig. S1**, **Movie S3-S6**). Concentrations chosen for ethanol were based on previously established dosage amounts utilizing NGM plates [50, 53]. Nicotine concentrations were chosen by evaluating the wide ranges of nicotine concentrations previously utilized for NGM studies. Prior studies have used a range of very low concentrations when assaying genetic and molecular targets of nicotine [54, 55]. In order to visualize phenotypic effects of nicotine utilizing the WormPharm optics, moderate to high concentrations were utilized to observe the more observable depressive effects previously seen in NGM studies. *C. elegans* demonstrated variable responses to nicotine and ethanol. The control worms, grown in untreated CeHR, developed into adults, evidenced by the detection of offspring in about four days, as expected. In the presence of varying concentrations of nicotine, the nematodes showed a remarkable decline in locomotion. Movement in the presence of 1 mM nicotine is characterized by a twitching motion, which persisted for about three days. Afterwards, an increase in thrashing and a decrease in twitching was observed. Development was delayed and only a few offspring were detected on the fourth day (**Fig. 5**, **Fig. S1**). Animals treated with > 1 mM concentrations of nicotine produced no offspring by the fourth day. In 10 mM nicotine, worms displayed a greater slowing down of movements accompanied by a more significant developmental phenotype. Interestingly, development and locomotion were affected more so in some than others, suggesting that some animals can develop resistance or tolerance to the nicotine. Treatment with 20 mM nicotine caused immediate paralysis followed by lethality by the second day. The slowed locomotion and depressed fertility of the animals have also been observed in NGM raised animals [51, 54]. We examined the growth dynamics of *C. elegans* in the presence of 1 mM and 10 mM nicotine using the WormPharm (**Fig. 4**). In 1 mM nicotine, the growth rate decreased significantly compared to untreated populations as expected, where the first wave of growth increase began to plateau at shortly after 4.5 days. A second wave of growth increase was not observed in the course of six days, indicating that reproduction was severely deterred. In 10 mM nicotine, the growth kinetics fluctuated close to baseline for about 2.5 days. Afterwards, the animals displayed almost no movement and development, causing the growth to remain static for the remainder of the experiment.

Ethanol at 200 mM and 400 mM concentrations induced more moderate effects compared to nicotine (**Fig. 5**; **Fig. S1**). In 200 mM ethanol, animals displayed no significant difference in locomotion or rate of development compared to the control animals. However, while eggs were observed by the fourth day, only a few L1 offspring were observed, suggesting an embryo-lethal phenotype. In 400 mM ethanol, development and locomotion were slightly reduced, and no offspring were detected on the fourth day. This slight delay in egg laying, along with the decrease in viable L1 animals in response to increasing concentrations of ethanol were also detected in animals exposed on NGM [52]. Our results indicate that CeHR grown *C. elegans* exposed to both of these drugs undergo similar effects when compared to those raised on NGM.

To assay the effects of co-administration of nicotine and ethanol, we selected moderate concentrations of each drug in order to observe the effects. In the presence of both 1 mM nicotine and 200 mM ethanol, the nematodes exhibit a substantial decrease in locomotion and a severe developmental phenotype (**Fig. 5**, **Fig. S1**). While most of the animals were viable throughout the experiment, growth did not persist after L1 stage of development. These observations suggest that nicotine and ethanol induce a very potent synergistic effect on *C. elegans* health. The reasons behind this synergistic effect are not yet known and require further investigation. It may be the case that the increased solubility of nicotine in ethanol enhances nicotine uptake by the worms.

### ChIP and chips: sequence data quality control and analysis

Epigenetic changes alter gene expression in response to environmental stimuli without changing the sequence of DNA [32, 33]. Epigenetics can be altered through a number of biological elements including: microRNAs, small RNAs, prions, DNA methylation and histone modifications. Histone modifications occur by post transcriptional chemical modification of the histone tails and act in diverse biological processes such as transcriptional activation/inactivation, chromosome packaging, and DNA damage/repair. To date, ChIP-seq is the most effective method to study how histone modifications can alter an organism’s epigenome.

**Figure 6:**
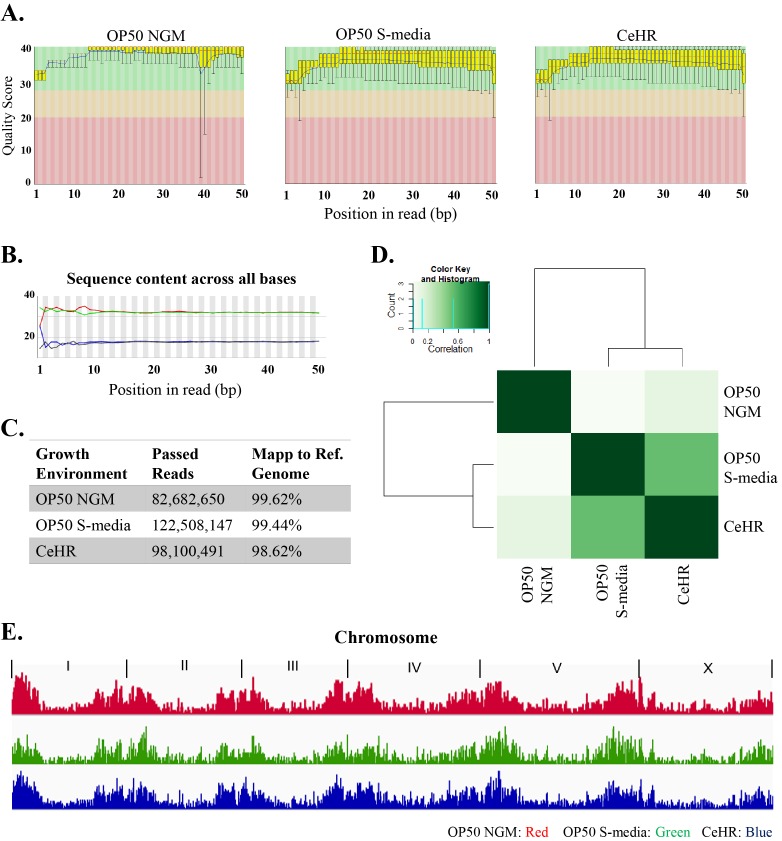
**Quality control and analysis of ChIP-seq data obtained from *C. elegans* grown on OP50 NGM, OP50 S-media and CeHR**. **A**. Quality scores of the ChIP sequence reads across all bases divided into three quality sections: very good (green), reasonable (orange), and poor (red). **B**. The percentages of the bases in each position of the total reads. **C**. Number and percentage of reads successfully mapped to the reference genome. **D**. Hierarchical clustering of the H3K27Me3 profiles across the genome for three different growth conditions. **E**. Visual comparison of genome-wide H3K27Me3 peaks for OP50 NGM, OP50 S-media and CeHR growth conditions.

We performed ChIP-seq to identify the distribution of trimethylated histone H3 on lysine 27 (H3K27me3) in *C. elegans* grown in Wormchips supplied with (1) OP50 NGM agar, (2) CeHR and (3) *E. coli* supplied liquid media (OP50 S-media). H3K27me3 is one of the best-known histone modifications in terms of both its biogenesis and effects, and the effect of H3K27me3 has been shown to be strongly repressive [56, 57]. We assessed the quality of all three ChIP sequences using the FastQC software [36]. **Figure 6A** represents an overview of the range of quality values across all bases. Sequences from all three growth conditions fall in the very good quality score regions (**Fig. 6A**). Along with the overall quality scores assigned to the reads, the following quality parameters were assessed: (1) per base sequence quality (2) *per se* quence quality scores, (3) per base sequence content, (4) per base GC content, (5) *per se* quence GC content, (6) per base N content, where N represents a substitution in case of failing to call a base with sufficient confidence, (7) sequence length distribution, (8) sequence duplication levels (overrepresented sequences) and (9) k-mer content. The three ChIP sequence datasets passed all quality parameters, except the “per base sequence content” (**Fig. 6B**). This failure occurs when any base (A, T, G, or C) is greater than 20% in any position, and this was expected in our analysis since the *C. elegans* genome has approximately 36% GC content [58].

Next, we mapped the ChIP sequence reads to the *C. elegans* reference genome (WBCel235) and acquired > 98% mapping scores for all three datasets (**Fig. 6C**). The reads were then used to identify regions in the genome (peaks) that were bound by H3K27Me3. The peaks were visualized by the Integrative Genomics Viewer to observe the overall differences in the distribution of H3K27Me3 between different growth conditions (**Fig. 6E**). Gene annotations were assigned to peak distributions to identify the associated genes. We categorized the genes that were differentially regulated in the various growth conditions, such as diet (CeHR vs. OP50) and physical environment (solid vs. liquid). We selected three representative genes for each growth condition or a combination of growth conditions (**Table 1**). For example, genes involved in the reproduction and muscle development were identified in animals grown on OP50 NGM but not in CeHR. Genes involved in neuronal functions were differentially regulated in animals grown in liquid (OP50 S-media and CeHR) but not on the solid (OP50 NGM) environments. In order to quantitatively compare the H3K27Me3 profiles, we created a hierarchical cluster (**Fig. 6D**). Worms grown in liquid OP50 S-media and CeHR have more similar H3K27Me3 distributions than worms cultured on OP50 NGM agar, suggesting that more epigenetic variations arose due to the difference in the diet rather than the physical environment.

## DISCUSSION

The WormPharm overcomes the limitations presented from bacterial cultivation by exploiting the axenic media, CeHR. The use of CeHR enables culturing of *C. elegans* for multiple generations over several months, which is unattainable with monoxenic cultivation. We also demonstrated the utility of the WormPharm to examine the effects of two psychoactive drugs, nicotine and alcohol on development, fecundity and growth dynamics. It is important to note that drug studies are best performed via axenic cultivation because bacteria can metabolize the drugs and cause variability in the assays. Furthermore, the WormPharm can autonomously culture *C. elegans* in six Wormchips, which enables experiments to be multiplexed with relative ease.

We observed that CeHR grown animals are phenotypically different from animals grown on OP50 NGM. These changes may be brought on by the differences in locomotion on solid versus liquid environments. Swimming and crawling are characterized by distinct kinematics and different underlying patterns of neuro-muscular activity in *C. elegans* [59], which can inflict diverse effects on energy consumption, muscle activities and behavior. In light of this, we compared animals grown on OP50 NGM and in OP50 S-media, where the only difference in the environment was the physical state. We observed only slight differences in body morphology, development and fecundity, suggesting that the liquid environment was not sole cause of the phenotypes observed in CeHR grown animals. Numerous lines of evidence have suggested that phenotypes induced by axenic cultivation are caused by the differences in the diet [13, 14, 16, 17, 46, 60-63]. Furthermore, we observed that the natural isolate AB1 *C. elegans* develops quicker and exhibits exaggerated morphological phenotypes, *i.e.* thinner and longer, compared to N2 animals in CeHR. These differences between AB1 and N2 animals suggest that the changes in body morphology may lead improved developmental success in *C. elegans*. Several studies have suggested that the laboratory domestication of the N2 *C. elegans* has given rise to genetic bottlenecks or laboratory selections [64, 65]. The selective pressures of unnatural environments may have limited the animals’ ability to adapt to axenic media. It is not fully understood what genetic and molecular-level changes are associated with diet or dietary restrictions in *C. elegans* [66], and we are currently conducting genome-wide and molecular analyses to better understand the effects of CeHR on *C. elegans* biology. Once these biological changes are identified, CeHR grown *C. elegans* may be used as a baseline for investigations on diet, dietary restrictions, aging, development, and pharmacology.

**Table 1:** **Short list of *C. elegans* genes associated with H3K27Me3 in different growth environments**. OP50 S-media and CeHR growth conditions are grouped as one category to portray genes bound to H3K27Me3 in animals grown in liquid environments but with a different diet. Conversely, OP50 S-media and OP50 NGM growth conditions portray genes bound to H3K27Me3 in animals grown in different physical environments but with the same diet.

Growth conditions	Gene	Human ortholog	Function
CeHR	smp-1	SEMAGC	Encodes a semaphorin; activity is required for vulval morphogenesis.
	rpa-0	RPLP0	Nucleic acid binding; involved in embryo development ending in birth or egg hatching.
	aak-1	PRKAA1	Required for negative regulation of germline proliferation during dauer development.
OP50 S-media	nfm-1	NF2	Structural constituent of cytoskeleton
	kap-1	KIFAP3	Microtubule motor activity
	msh-2	MSH2	Encodes a DNA mismatch repair protein
OP50 S-media and CeHR	ketn-1	MYPN	Involved in response to hypoxia and expressed in the reproductive system and several muscle types.
	dhs-6	HSDL2	Oxidoreductase activity
	tsp-14	TSPAN8	Receptor binding
OP50 S-media and NGM	tmd-2	LMOD2	Actin binding; predicted to have tropomyosin binding activity.
	rps-20	RPS20	Encodes a small ribosomal subunit S20 protein.
	T12C9.7	CCNO	Kinase activator activity

Our ChIP-seq analysis of *C. elegans* cultured in the WormPharm identified an array of epigenetic variations that arose from the animals’ response to different growth conditions. We want to emphasize that the number of *C. elegans* cultured in a single CeHR supplied Wormchip was sufficient to perform ChIP-seq targeting at least one histone modification, which was unobtainable with a similar volume of OP50 S-media. Furthermore, the ChIP sequence data quality was very high. Since the purpose of this study is solely to demonstrate the utility of the WormPharm to culture *C. elegans* for genomic studies, only one ChIP-seq assay per growth condition is represented. Typically, at least two replicates are required to acquire significant and conclusive assessments from ChIP-seq analyses due to the intrinsic variability of the assay. In light of this, we will perform the necessary technical and biological replicates and downstream analyses to characterize epigenetic alterations in *C. elegans* under these different growth conditions; however, it is outside the scope of this paper and will be discussed in a separate study.

The WormPharm was originally designed as an automated *C. elegans* culturing system for space biology studies. As humans begin an era of space exploration, it will be important to understand how biology reacts to microgravity and long duration space flight. *C. elegans* is an ideal organism to study these effects as *C. elegans* homologues have been identified for 60–80% of human genes [67, 68]. *C. elegans* also bridges the gap between cell cultures and mammalian models because it allows for large population studies unattainable with small mammals. However, there are limitations to the canonic method of *C. elegans* cultivation (OP50 NGM) in space because closed environments like the International Space Station (ISS) are easily contaminated, which can compromise other experiments. Thus, replenishment of the bacterial food source is not a preferred option for research aboard space flight missions. The WormPharm enables longer-term and multigenerational studies to be conducted by feeding nematodes axenic media in a sterile and completely sealed fluid pump system. Furthermore, the WormPharm can be used to perform multiple experiments with minimal crew time due to its automated and multiplexed design. The WormPharm can monitor and score organismic phenotypes and growth kinetics while in space and also provide for the opportunity to perform genome-wide analyses once the WormPharm and its cargo returns to Earth. Lastly, the WormPharm will be a powerful tool to screen for drug candidates that can mitigate health risks due to microgravity such as bone and muscle loss, and loss of hydrostatic pressure of fluids inside the body. To date, the longest spaceflight involving *C. elegans* was for six months in low Earth orbit onboard the ISS [69, 70]. Szewczyk and colleagues utilized the chemically defined CeMM, which is similar in chemical composition to CeHR, but without skim milk. The study used CeMM in conjunction with a remote automated culturing system consisting of pumps and video capturing devices. The small footprint of our WormPharm can potentially be useful on miniaturized satellites, *i.e.* micro- or nano-satellites. Such biosatellites can enable space biology research beyond low Earth orbit, opening more doors to a new realm of space research, particularly with respect to galactic cosmic radiation and solar proton events of deep space.

## Abbrievations used

NGM: Nematode Grown Medium
E. coli: Escherichia coli
CeHR: *C. elegans* Habitation and Reproduction medium
CeMM: C. elegans Maintenance Medium
ChIP-seq: chromatin-immunoprecipitation followed by next generation sequencing
Nic: nicotine
EtOH: ethanol
DNA: deoxyribonucleic acid
H3K27me3: trimethylated histone H3 on lysine 27
ISS: International Space Station

## Supplementary information

**Figure S1**. *C. elegans* display aberrant growths in response to nicotine and alcohol

**Movie S1**. N2 on OP50 NGM Agar Growth Kinetics

**Movie S2**. N2 in CeHR Growth Kinetics

**Movie S3**. N2 in CeHR on Day 6

**Movie S4**. N2 in 10 mM Nicotine Day 6

**Movie S5**. N2 in 400 mM EtOH Day 6

**Movie S6**. N2 in 1 mM Nicotine and 200 mM EtOH Day 6
